# Prediction of collateral circulation grading and functional outcomes in acute ischemic stroke using FLAIR vascular hyperintensity combined with multimodal CT parameters

**DOI:** 10.3389/fneur.2026.1688188

**Published:** 2026-01-29

**Authors:** Qiushi Yang, Yanxin Ma, Xin Qu, Yu Chen, Hui Sun, Gang Yao

**Affiliations:** 1 Mudanjiang Medical University, Mudanjiang, Heilongjiang, China; 2 Department of Radiology, Daqing Oilfield General Hospital, Daqing, Heilongjiang, China

**Keywords:** collateral circulation, fluid-attenuated inversion recovery vascular hyperintensity, functional outcome, ischemic stroke, prediction model

## Abstract

**Background/objectives:**

The variability in acute ischemic stroke (AIS) outcomes is closely associated with collateral circulation status. While fluid-attenuated inversion recovery vascular hyperintensity (FVH) and multimodal CT parameters (e.g., rLMC score, rCBV) were associated with 90-day functional outcomes in AIS patients, their combined predictive value and clinical utility warrant further investigation. This study investigates the combined predictive value of FVH and multimodal CT parameters for collateral assessment and prognosis in AIS.

**Methods:**

We retrospectively and consecutively enrolled AIS patients with internal carotid artery or middle cerebral artery stenosis/occlusion who did not receive intravenous thrombolysis or mechanical thrombectomy. All patients underwent one-stop CT angiography–CT perfusion and multimodal MRI within 72 h of symptom onset. Evaluations included FVH scores (based on modified ASPECTS regions), rLMC scores, Maas scores, and ASITN/SIR collateral grading. Spearman analysis assessed correlations between FVH and CTA collateral scores. Univariate and multivariate logistic regression indicated the independent predictors of a 90-day functional outcome [favorable (mRS 0–2) vs. poor (mRS 3–6)], with receiver operating characteristic (ROC) curves evaluating predictive performance.

**Results:**

The cohort comprised 112 patients (70 favorable outcomes, 42 poor outcomes). FVH scores showed a negative correlation with ASITN/SIR collateral grades (*r* = −0.432, *p* < 0.001). Compared to the favorable outcome group, the poor outcome group exhibited higher baseline National Institute of Health Stroke Scale (NIHSS) scores, elevated FVH scores, reduced rLMC scores, and lower rCBV values (all *p* < 0.05). Multivariate analysis indicated that NIHSS score, FVH score, rLMC score, and rCBV were independent predictors of poor outcomes. ROC analysis demonstrated strong predictive performance for rLMC score (AUC = 0.848, 95%CI 0.778–0.919), FVH score (AUC = 0.662, 95%CI 0.550–0.774), and rCBV (AUC = 0.727, 95%CI 0.631–0.822).

**Conclusion:**

Multimodal CT combined with MRI facilitates early AIS diagnosis and collateral assessment. The integration of FVH with CT parameters (rLMC score and rCBV) was associated with the prediction of functional outcomes in AIS patients.

## 1 Introduction

A Global Burden of Disease (GBD) study revealed a substantial annual increase in stroke incidence and mortality, establishing stroke as the world’s second-leading fatal disease after cardiovascular disease, characterized by its high morbidity, disability rate, and mortality. Among all stroke subtypes, acute ischemic stroke (AIS) caused by large- or medium-artery stenosis or occlusion is the most prevalent (1).

Clinical studies have observed significant variability in neurological recovery and long-term outcomes among AIS patients with similar degrees of intracranial large-artery stenosis or occlusion, even after receiving thrombolysis or mechanical thrombectomy (2–5). Emerging evidence suggests that collateral circulation formation and compensatory capacity are critical determinants of ischemic penumbra survival and clinical prognosis (6). When blood flow is interrupted due to arterial occlusion or stenosis, pre-existing anastomotic vessels dilate or form capillary connections under hypoperfusion stress, establishing microcirculatory bypasses that redirect blood flow to ischemic regions (7).

Conventionally, collateral status is assessed using computed tomography angiography (CTA)-based scoring systems. CTA offers a non-invasive, cost-effective, and reproducible method for collateral evaluation, providing multimodal information, which is crucial for AIS diagnosis, treatment selection, and prognosis (8).

In 1999, Cosnard et al. (9) first described fluid-attenuated inversion recovery vascular hyperintensity (FVH) on MRI-FLAIR sequences—a serpentine hyperintensity along cortical vessels, typically in the frontal or temporal lobes. FVH likely reflects slow anterograde or retrograde collateral flow distal to stenotic/occluded vessels due to diminished flow-void effects, serving as a non-contrast marker of leptomeningeal collaterals in early AIS. Studies present conflicting findings: Lyu et al. (10) identified FVH as an independent predictor of poor functional recovery, suggesting its association with inadequate collaterals, whereas Xin et al. (11) correlated higher FVH scores with robust collateral grades and favorable outcomes.

Although multimodal MRI remains sensitive for early infarct detection, prolonged scan times and motion artifacts limit its utility. Conversely, one-stop CT angiography —CT perfusion (P) provides rapid, comprehensive hemodynamic assessment but may miss early ischemic changes. Combining these modalities has the potential to improve prognostic assessment by evaluating collateral status and perfusion dynamics (12).

## 2 Materials and methods

### 2.1 Study design and participants

We conducted a retrospective analysis of 224 AIS patients admitted to the Department of Neurology, Daqing Oilfield General Hospital, from May 2023 to December 2024. After strict screening per the inclusion/exclusion criteria, 112 patients were enrolled. The study protocol was approved by the Institutional Review Board of Daqing Oilfield General Hospital (Approval No. ZYAF/SC--07/02.0), and written informed consent was obtained from all participants in strict accordance with the Declaration of Helsinki ethical guidelines.

### 2.2 Clinical data collection

We collected comprehensive baseline clinical data from all enrolled patients, such as gender, age, admission-to-examination time interval, systolic and diastolic blood pressure, heart rate, stroke risk factors (hypertension, diabetes mellitus, hyperlipidemia, coronary artery disease, atrial fibrillation, previous stroke history), location of arterial stenosis or occlusion, medication use and serological biomarkers [total cholesterol (TC), triglycerides (TG), high-density lipoprotein (HDL), low-density lipoprotein (LDL), homocysteine (HCY), uric acid (UA), blood glucose (BS), and glycated hemoglobin (HbA1c)]. Neurologists assessed National Institutes of Health Stroke Scale (NIHSS) scores on admission day. The assessors were blinded to the patients’ imaging findings. The modified Rankin Scale (mRS) was used to evaluate 90-day clinical outcomes, with patients categorized into favorable outcome (mRS 0–2) and poor outcome (mRS 3–6) groups.

#### 2.2.1 Inclusion criteria

1. Age ≥18 years.
2. Time from symptom onset to completion of one-stop CTA–CTP and multimodal MRI ≤ 72 h.
3. Diagnosis of AIS confirmed by DWI or CTA, with severe stenosis (≥70%) or occlusion of the internal carotid artery (ICA) and/or middle cerebral artery (MCA).
4. Received standard medical therapy (antiplatelet/anticoagulant, statins, and risk factor control).

#### 2.2.2 Exclusion criteria

1. Intracranial hemorrhage, tumor, trauma, or other neurological disorders on CT/MRI.
2. Posterior circulation or bilateral infarcts.
3. Prior intravenous thrombolysis or mechanical thrombectomy.
4. Contraindications to CT/MRI.
5. Incomplete imaging/clinical data or poor image quality.

**Figure 1:**
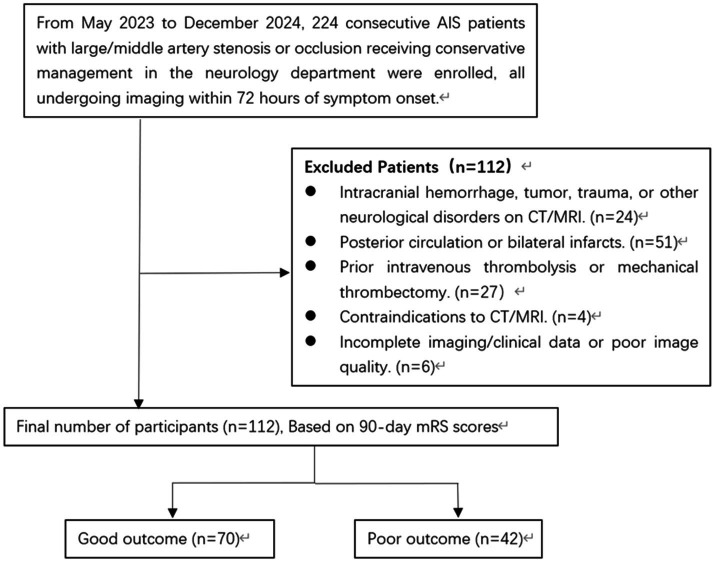
Flow diagram of enrollment of study patients.

This retrospective cohort study initially screened 224 consecutive acute ischemic stroke (AIS) patients with large- or middle-cerebral artery occlusion who did not receive reperfusion therapy. After the sequential exclusion of 112 patients (50%) for specific reasons—such as comorbid neurological conditions (*n* = 24), posterior circulation or bilateral infarcts (*n* = 51), prior thrombolysis/thrombectomy (*n* = 27), contraindications to imaging (*n* = 4), and incomplete/poor-quality data (*n* = 6)—a final cohort of 112 patients was constituted. These 112 patients were subsequently stratified into a good outcome group (*n* = 70) and a poor outcome group (*n* = 42) based on their 90-day functional status (modified Rankin Scale score; Figure 1).

### 2.3 Imaging protocol

All patients underwent one-stop CTA–CTP scanning using the United Imaging uCT960 + system and MRI examination with diffusion-weighted imaging (DWI) sequences performed on a GE 1.5 T HD-xt scanner equipped with an eight-channel head coil. Prior to imaging, an intravenous catheter was placed in the right median cubital vein, and patients received standardized instructions regarding examination procedures and potential physiological reactions during scanning.

All patients underwent one-stop CT scanning using the United Imaging uCT960 + 640-slice CT scanner (320 detector rows), which included sequential non-contrast CT, CT perfusion (CTP), and CT angiography (CTA) scans. The non-contrast CT parameters were set at 120 kV tube voltage, 300 mA tube current, with 5-mm slice thickness and 5-mm slice interval. For CTP scanning, the parameters were 100 kV tube voltage, 120-mA tube current, 5-mm slice thickness and interval, 1.0-s rotation time, using a power injector to administer 50 mL of non-ionic iodinated contrast (Iohexol, GE Healthcare, 350 mgI/mL) followed by 30 mL saline flush at 5 mL/s injection rate through the right median cubital vein, with a 5-s delay before initiating scanning over a 160-mm range from skull base to vertex, acquiring 19 dynamic scans. The acquired data were reconstructed into 5-mm thick slices with 5-mm intervals for CTP analysis and 1.5 mm slice width for multiphase CTA (mCTA) analysis, with mCTA images generated by post-processing the original CTP data using the United Imaging CT workstation. Multimodal MRI was performed on a GE 1.5 T HD-xt system (GE Healthcare, United States) with the following protocol: T1-weighted imaging (TR/TE = 1,625/24 ms, FOV 240 × 240 mm, 6-mm slice thickness, acquisition time 1 min 14 s), T2-weighted imaging (TR/TE = 4,949/108.7 ms, FOV 240 × 240 mm, 6-mm slice thickness, 45 s acquisition), diffusion-weighted imaging (TR/TE = 4,053/84.2 ms, b-value = 1,000 s/mm^2^, FOV 240 × 240 mm, 6-mm slice thickness, 32 s acquisition), and FLAIR imaging (TR/TE = 8,400/100 ms, 5-mm slice thickness, FOV 240 × 240 mm, 2 min 23 s acquisition).

### 2.4 Image analysis

All images were independently evaluated by two experienced neuroradiologists blinded to clinical data, with slice-by-slice analysis of lesions across all sequences. Discrepancies were resolved through consensus discussion.

CTP raw data were processed using uAI Discover–Cerebral Perfusion V1.0 (United Imaging Intelligence, China), with the contralateral ICA/MCA origin manually marked as the input artery and the superior sagittal sinus as the output vein to generate time-density curves (TDC). Manual correction was applied when TDC abnormalities resulted from incorrect vessel selection. The software automatically produced color-coded perfusion maps (CBF, CBV, MTT, TTP, and Tmax) and a bone-removed CTA-MIP image from the optimal phase. CTA images were reconstructed into MPR, MIP, and VR formats. Multiphase CTA data were analyzed using FastStroke software, with two neuroradiologists assessing collateral circulation status on triphasic CTA-MIP and sCTA-MIP images using the contralateral hemisphere as reference.

All imaging analyses were performed using standardized scoring systems. The Alberta Stroke Program Early CT Score (ASPECTS) was applied to DWI images, dividing the MCA territory into 10 regions across nuclear and supra-nuclear levels: M1 (anterior MCA cortex); M2 (lateral insula); M3 (posterior MCA cortex) at the nuclear level; M4–M6 (anterior, lateral and posterior MCA cortices) at the supra-nuclear level; plus caudate head (C), lentiform nucleus (L), internal capsule posterior limb (IC), and insula (I). Scores range from 10 (no infarction) to 0 (complete MCA territory infarction) (13).

**Figure 2:**
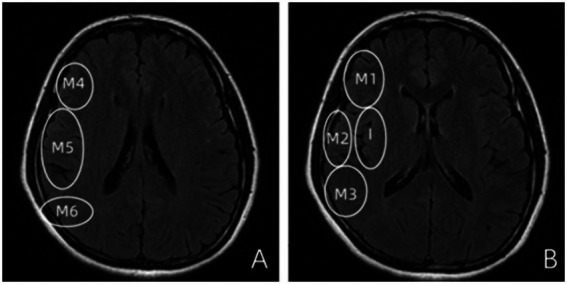
Schematic illustration of the FLAIR vascular hyperintensity (FVH) scoring regions based on the modified Alberta Stroke Program Early CT Score (ASPECTS) template. **(A)** Depicts the lateral cortical regions of the middle cerebral artery (MCA) territory: M4 (anterior frontal cortex), M5 (lateral frontal cortex), and M6 (posterior frontal or anterior parietal cortex). **(B)** Shows the insular region (I) and the remaining cortical regions: M1 (anterior temporal cortex), M2 (lateral temporal cortex), and M3 (posterior temporal or temporo-occipital cortex). This seven-region scoring system (I, M1–M6) is used for the semiquantitative assessment of FVH burden, with each region exhibiting FVH assigned 1 point (total score range: 0–7).

Fluid-attenuated inversion recovery vascular hyperintensities (FVH) were defined as serpentine, tubular, or dot-like hyperintensities along the cortical vessels on FLAIR sequences, typically distal to the site of arterial occlusion or stenosis. FVH was rated using a semiquantitative scoring system adapted from the Alberta Stroke Program Early CT Score (ASPECTS), known as the FVH-ASPECTS score. This system divides the middle cerebral artery (MCA) territory into seven regions: the insular ribbon (I) and the M1–M6 cortical regions. Each region exhibiting a positive FVH sign was assigned 1 point. The total FVH score thus ranged from 0 to 7, with a higher score indicating a more extensive FVH burden (14) (Figure 2).

The Maas collateral score evaluated sylvian fissure and cortical collateral filling: 1 = absent; 2 = reduced; 3 = equal; 4 = greater; 5 = complete collateral flow. Scores 1–2 indicated poor and 3–5 good collaterals (15).

The regional leptomeningeal collateral (rLMC) score assessed collateral filling in ASPECTS regions (M1-M6), ACA territory, basal ganglia, and sylvian fissure: 0 = no filling; 1 = reduced; 2 = equal/greater (sylvian fissure scored 0/2/4). Total scores 0–10 indicated poor, and 11–20 good collaterals (16).

**Figure 3:**
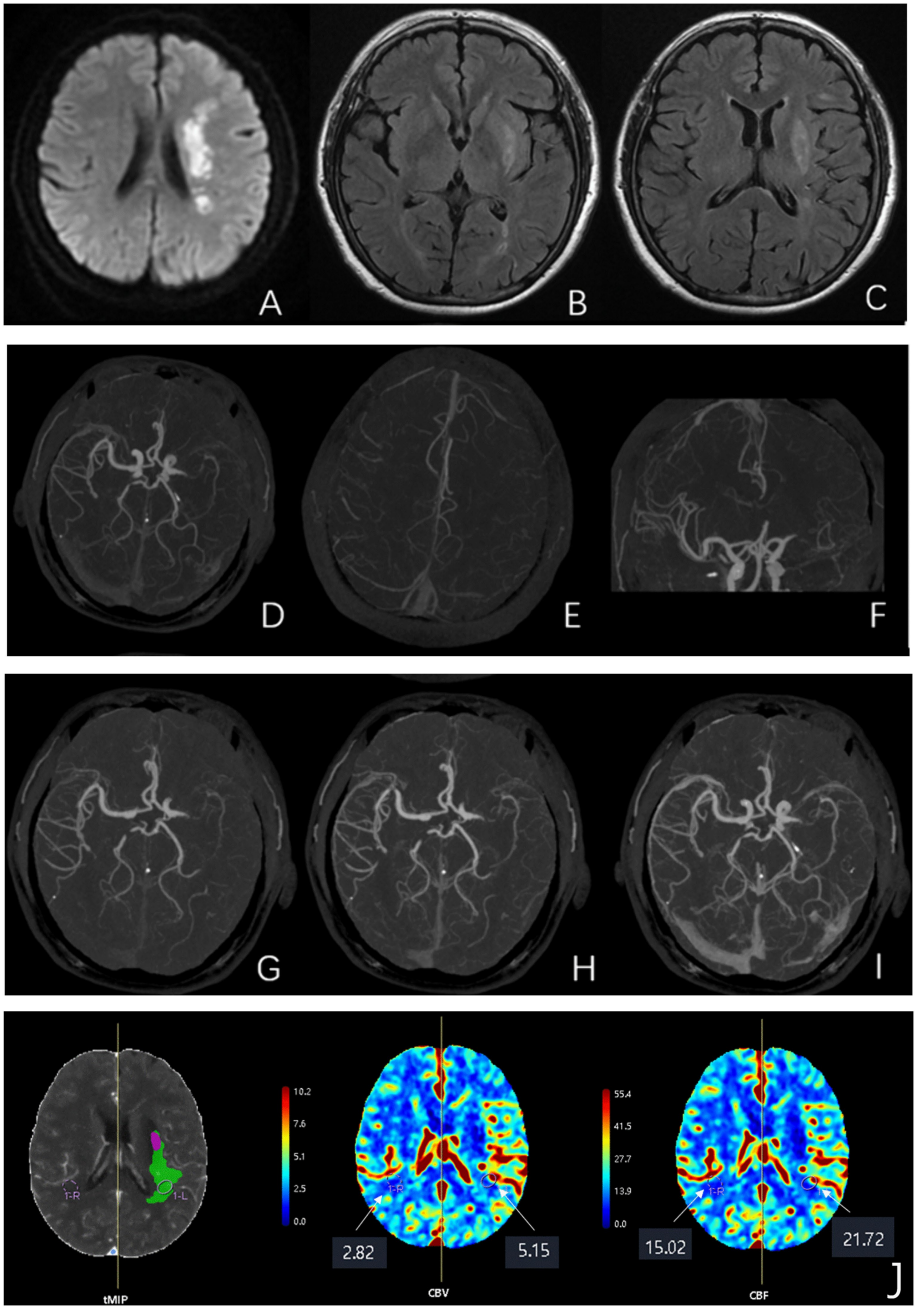
Multimodal neuroimaging findings in a representative patient with left M1 segment occlusion and a 90-day mRS score of 2. Panel A: DWI and FLAIR sequences. **(A)** An axial diffusion-weighted imaging (DWI) scan demonstrates an acute ischemic infarction (hyperintense signal) in the left middle cerebral artery (MCA) territory. **(B,C)** Concomitant axial T2-weighted fluid-attenuated inversion recovery (FLAIR) images reveal prominent FLAIR vascular hyperintensity (FVH) signs (serpentine hyperintensities, indicated by arrows) along the cortical sulci, suggestive of slow anterograde collateral flow. Panel B: Structural angiography (sCTA). **(D–F)** Single-phase spiral CT angiography (sCTA) source images confirm the occlusion at the proximal M1 segment of the left MCA (arrowheads), with an abrupt cut-off of contrast opacification. Panel C: Hemodynamic angiography (mCTA). **(G–I)** Reconstructed multiphase CT angiography (mCTA) images (time-resolved) illustrate the dynamics of collateral circulation. The delayed and partial retrograde filling of the distal MCA branches (arrows) indicates inadequate leptomeningeal collateral compensation, correlating with persistent perfusion deficits. Panel D: Perfusion parameter maps. **(J)** Quantitative perfusion maps derived from CT perfusion (CTP) data. Compared to the contralateral hemisphere, the affected left hemisphere shows elevated cerebral blood flow (CBF, green overlay) and cerebral blood volume (CBV, purple overlay), indicating a mismatch consistent with the ischemic penumbra. MCA, middle cerebral artery; DWI, diffusion-weighted imaging; FVH, FLAIR vascular hyperintensity; sCTA, spiral CT angiography; mCTA, multiphase CT angiography; CBV, cerebral blood volume; CBF, cerebral blood flow; MCA, middle cerebral artery; mRS, modified Rankin Scale.

The ASITN/SIR collateral grading system (modified from multiphase CTA-DSA) classified collateral flow as: 0 = no collaterals; 1 = slow peripheral filling with persistent defect; 2 = rapid peripheral filling with partial ischemic zone perfusion; 3 = slow but complete late venous filling; 4 = complete rapid retrograde perfusion. Grades 0–2 indicated poor, and 3–4 good collaterals (17). Figure 3 shows typical serial images.

### 2.5 Statistical analysis

Statistical analyses were performed using R software (version 4.2.0). Continuous variables with normal distribution were expressed as mean ± standard deviation (SD), and group comparisons were conducted using Student’s *t*-test. For non-normally distributed continuous variables, data were presented as median (interquartile range, IQR), and the Mann–Whitney *U-*test was applied for group comparisons. Categorical variables were described as frequencies (percentages), and differences between groups were assessed using the chi-squared test or Fisher’s exact test, as appropriate.

Inter-rater reliability for CT- and MRI-based collateral circulation scoring scales was evaluated using Cohen’s kappa (*κ*) statistics. Spearman’s rank correlation analysis was performed to assess the association between CTA/MRI-derived collateral scores and the ASITN/SIR collateral grading system (modified from multiphase CTA-DSA).

Univariate analysis followed by multivariate binary logistic regression was used to identify independent predictors of patient prognosis (favorable vs. poor outcome). The final multivariate model included variables with *p* < 0.1 from univariate analysis, following confirmation of no severe multicollinearity (all VIFs <5). Data are shown as adjusted ORs (95% CIs). The receiver operating characteristic (ROC) curve analysis was employed to evaluate the predictive performance of collateral circulation scores for clinical outcomes in conservatively treated patients. A two-tailed *p*-value <0.05 was considered statistically significant.

## 3 Results

### 3.1 Clinical outcomes

A total of 224 consecutive patients with acute ischemic stroke and confirmed large- or middle-cerebral artery occlusion, who did not receive reperfusion therapy, were initially screened. After applying the predefined exclusion criteria (detailed in the patient flow diagram, Figure 1), 112 patients (50%) were excluded, resulting in a final study cohort of 112 patients. These patients were dichotomized based on their 90-day modified Rankin Scale score into a good functional outcome group (mRS 0–2, n = 70) and a poor functional outcome group (mRS 3–6, n = 42). The median time from symptom onset to imaging was 20 h (IQR 6.0–30.0). Patients had a median age of 63 years (IQR 55–70), with 83 men (74%) and 29 women (26%). Comorbidities were prevalent: hypertension in 103 patients (92%), diabetes mellitus in 58 (52%), hyperlipidemia in 46 (41%), coronary artery disease in 26 (23%), atrial fibrillation in 7 (6.3%), and a previous stroke history in 29 (26%). Medication use at admission was as follows: antihypertensive drugs in 103 (92.0%), lipid-lowering drugs in 107 (95.5%), antiplatelet agents in 103 (92.0%), and drugs for microcirculation improvement in 75 (67.0%). Admission vital signs demonstrated a median systolic blood pressure of 146 mmHg (IQR 134–165), diastolic pressure of 88 mmHg (IQR 77–96), and heart rate of 77 beats per minute (IQR 70–88). Laboratory parameters at admission included a median blood glucose level of 6.1 mmol/L (IQR 4.9–9.6), glycated hemoglobin of 6.43% (IQR 6.16–7.75), homocysteine of 11.5 μmol/L (IQR 9.4–14.7), total cholesterol of 4.24 mmol/L (IQR 3.57–5.10), triglycerides of 1.58 mmol/L (IQR 1.10–2.31), high-density lipoprotein of 0.96 mmol/L (IQR 0.82–1.12), low-density lipoprotein of 2.66 mmol/L (IQR 2.05–3.22), and uric acid of 307 μmol/L (IQR 251–354). Vascular imaging revealed internal carotid artery stenosis in 35 patients (31.3%), middle cerebral artery stenosis in 70 (62.5%), and combined lesions in 7 (6.3%). Neurological assessment showed a median NIHSS score of 3 (IQR 2–6) and DWI-ASPECTS of 7 (IQR 6–8) at admission (Table 1).

**Table 1:** Comparisons of clinical and imaging characteristics in patients with good and poor outcome.

Variable	Total (*n* = 112)	Good prognosis (*n* = 70)	Poor prognosis (*n* = 42)	Statistic	*p*-value
Age	63 (55, 70)	62.00 (53.25–69.00)	64.50 (59.50–71.50)	1.807	0.179
Sex				0.251	0.616
Male	83 (74%)	53 (75.71%)	30 (71.43%)		
Female	29 (26%)	17 (24.29%)	12 (28.57%)		
Onset-to-imaging time (h)	20.0 (6.0, 30.0)	20.0 (6.0, 30.0)	12.0 (4.0, 24.0)	1309.500	0.334
Hypertension				1.361	0.243
No	103 (92%)	66 (94.29%)	37 (88.10%)		
Yes	9 (8.0%)	4 (5.71%)	5 (11.90%)		
Diabetes				2.756	0.097
No	58 (52%)	38 (54.29%)	16 (38.10%)		
Yes	54 (48%)	32 (45.71%)	26 (61.90%)		
Hyperlipidemia				0.010	0.921
No	46 (41%)	29 (41.43%)	17 (40.48%)		
Yes	66 (59%)	41 (58.57%)	25 (59.52%)		
Coronary artery disease				2.257	0.133
No	26 (23%)	13 (18.57%)	13 (30.95%)		
Yes	86 (77%)	57 (81.43%)	29 (69.05%)		
Atrial fibrillation				1.229	0.268
No	105 (94%)	67 (95.71%)	38 (90.48%)		
Yes	7 (6.3%)	3 (4.29%)	4 (9.52%)		
Prior stroke history				0.698	0.403
No	29 (26%)	20 (28.57%)	9 (21.43%)		
Yes	83 (74%)	50 (71.43%)	33 (78.57%)		
Stenosis site				3.017	0.221
ICA	35 (31%)	26 (37.14%)	9 (21.43%)		
MCA	70 (63%)	40 (57.14%)	30 (71.43%)		
ICA + MCA	7 (6.3%)	4 (5.71%)	3 (7.14%)		
Medication use					
Antihypertensive drugs				1.361	0.243
No	9 (8.0%)	4 (5.7%)	5 (11.9%)		
Yes	103 (92.0%)	66 (94.3%)	37 (88.1%)		
Lipid-lowering drugs				0.691	0.408
No	5 (4.0%)	4 (5.7%)	1 (2.4%)		
Yes	107 (96.0%)	66 (94.3%)	41 (97.6%)		
Antiplatelet agents				0.201	0.654
No	9 (8.0%)	5 (7.1%)	4 (9.5%)		
Yes	103 (92.0%)	65 (92.9%)	38 (90.5%)		
Drugs for microcirculation improvement				1.682	0.195
No	37 (33.0%)	20 (28.6%)	17 (40.5%)		
Yes	75 (67.0%)	50 (71.4%)	25 (59.5%)		
Systolic BP (mmHg)	146 (134, 165)	147.23 ± 22.01	151.02 ± 23.02	0.754	0.387
Diastolic BP (mmHg)	88 (77, 96)	87.54 ± 13.32	83.40 ± 15.88	2.191	0.142
Heart rate (bpm)	77 (70, 88)	79.39 ± 12.12	78.76 ± 14.64	0.059	0.808
Total cholesterol (mmol/L)	4.24 (3.57, 5.10)	4.28 (3.74–5.11)	4.24 (3.43–4.97)	0.417	0.518
Triglycerides (mmol/L)	1.58 (1.10, 2.31)	1.61 (1.07–2.38)	1.53 (1.14–2.10)	0.206	0.650
HDL (mmol/L)	0.96 (0.82, 1.12)	0.94 (0.80–1.11)	0.99 (0.83–1.13)	0.283	0.595
LDL (mmol/L)	2.66 (2.05, 3.22)	2.72 ± 0.93	2.62 ± 0.85	0.299	0.586
Uric acid (μmol/L)	307 (251, 354)	306.00 (252.55–352.93)	310.00 (247.75–355.75)	0.073	0.787
Homocysteine (μmol/L)	11.5 (9.4, 14.7)	11.27 (9.55–13.83)	11.84 (9.10–15.05)	0.157	0.692
Blood glucose (mmol/L)	6.1 (4.9, 9.6)	5.80 (4.92–9.18)	6.91 (4.98–10.21)	1.388	0.239
HbA1c (%)	6.43 (6.16, 7.75)	6.31 (6.13–7.70)	6.90 (6.27–7.95)	3.192	0.074
NIHSS score	3.00 (2.00–6.00)	3.00 (1.00–4.00)	5.50 (3.00–10.00)	16.429	<0.001
DWI-ASPECTS	7.00 (6.00–8.00)	8.00 (7.00–8.00)	7.00 (6.00–8.00)	1.244	0.265
FVH score	2.00 (1.00–4.00)	1.00 (1.00–4.00)	3.00 (3.00–4.00)	8.425	0.004
rLMC score	13.00 (9.00–14.00)	14.00 (12.00–15.00)	9.00 (6.00–12.00)	38.257	<0.001
Maas score	3.00 (2.00–4.00)	3.00 (3.00–4.00)	2.00 (1.00–3.00)	25.798	<0.001
ASITN/SIR score	3.00 (2.00–4.00)	3.00 (3.00–4.00)	2.00 (2.00–3.00)	1.967	0.161
rCBV	1.06 (0.96, 1.26)	1.19 (1.01–1.36)	1.01 (0.91–1.08)	16.046	<0.001
rCBF	0.94 (0.82, 1.12)	0.99 (0.83–1.17)	0.90 (0.81–0.98)	4.707	0.030

ICA, internal carotid artery; MCA, middle cerebral artery; HDL, high-density lipoprotein; LDL, low-density lipoprotein; DWI-ASPECTS, diffusion-weighted imaging–Alberta Stroke Program Early CT Score; NIHSS, National Institutes of Health Stroke Scale; FVH, FLAIR vascular hyperintensity; rLMC, regional leptomeningeal collaterals; Maas, Maasricht Acute Stroke Score; ASITN/SIR, American Society of Interventional and Therapeutic Neuroradiology/Society of Interventional Radiology; rCBV, relative cerebral blood volume; rCBF, relative cerebral blood flow.

### 3.2 Collateral circulation scoring results in AIS patients

The evaluation of collateral circulation using various scoring systems demonstrated the following distribution among the 112 AIS patients:

For CTA-based collateral assessments, the Maas score classified 46 patients (41.1%) as having poor collateral circulation (scores 1–2) and 66 patients (58.9%) with good collateral circulation (scores 3–5). The rLMC scoring system identified 43 patients (38.4%) with poor collaterals (scores 0–10) and 69 patients (61.6%) with good collaterals (scores 11–20). When applying the ASITN/SIR grading system adapted from DSA standards, 40 patients (35.7%) showed poor collateral circulation (grades 0–2) while 72 patients (64.3%) exhibited good collateral circulation (grades 3–4).

The FVH scoring system, based on FLAIR MRI sequences, categorized patients into 57 cases (50.9%) with lower scores (0–3 points) and 49 cases (43.8%) with higher scores (4–7 points).

### 3.3 Inter-rater reliability of collateral circulation scores

The Kappa consistency analysis revealed good agreement between junior and senior radiologists across all three collateral scoring systems. The inter-rater reliability was highest for the rLMC score, followed by the FVH score, with the Maas score showing slightly lower but still acceptable consistency between raters (Table 2).

**Table 2:** Inter-rater reliability of collateral circulation scoring.

Measure	Kappa value	95% CI	*p*-value
FVH score	0.77	0.68–0.85	<0.001
Maas score	0.75	0.66–0.85	<0.001
rLMC score	0.79	0.71–0.87	<0.001

FVH, FLAIR vascular hyperintensity; Maas, Maastricht Acute Stroke Score; rLMC, regional leptomeningeal collateral score; CI, confidence interval.

### 3.4 Correlation analysis between collateral scores and ASITN/SIR grading system

Spearman correlation analysis demonstrated that rLMC score and Maas score were positively correlated with ASITN/SIR collateral grading, while FVH score showed a negative correlation (*p* < 0.001). The correlation coefficients, ranked from highest to lowest, were as follows: rLMC score > Maas score > FVH score (Table 3).

**Table 3:** Correlation analysis between collateral scores and ASITN/SIR grading system.

Measure	*r*-value	95% CI	*p*-value
FVH score	−0.59	−0.70 - -0.45	<0.001
Maas score	0.63	0.49–0.73	<0.001
rLMC score	0.84	0.78–0.89	<0.001

FVH, FLAIR vascular hyperintensity; Maas, Maastricht Acute Stroke Score; rLMC, regional leptomeningeal collateral score; CI, confidence interval.

### 3.5 Analysis of prognostic factors in AIS patients

There were 70 patients with favorable outcomes and 42 patients with poor outcomes. Univariate analysis of the overall sample showed that compared with patients having poor outcomes, those with favorable outcomes had significantly lower admission NIHSS scores (*p* < 0.05), significantly lower FVH scores (*p* < 0.05), significantly higher proportions of good collateral circulation status as indicated by rLMC and Maas scores (*p* < 0.05), and significantly higher rCBV and rCBF values (*p* < 0.05). No statistically significant differences were found between the two groups for all other indicators (all *p* > 0.05) (Table 4).

**Table 4:** Evaluation of factors associated with prognosis by univariate logistic regression analysis.

Variable	Good outcome (*N* = 70)	Poor outcome (*N* = 42)	OR (95% CI)	*p*-value
Sex			1.25 (0.53–2.96)	0.617
Male	53 (75.7%)	30 (71.4%)		
Female	17 (24.3%)	12 (28.6%)		
Age (years)	61.2 ± 11.8	64.1 ± 9.6	1.03 (0.99–1.06)	0.179
Onset-to-imaging time (h)	20.0 ± 15.6	18.4 ± 17.7	0.99 (0.97–1.02)	0.613
NIHSS score				
≤ 3.5	48 (68.6%)	11 (26.2%)		
> 3.5	22 (31.4%)	31 (73.8%)	6.15 (2.62–14.43)	<0.001
Hypertension				
No	4 (5.7%)	5 (11.9%)		
Yes	66 (94.3%)	37 (88.1%)	0.45 (0.11–1.77)	0.253
Diabetes				
No	38 (54.3%)	16 (38.1%)		
Yes	32 (45.7%)	26 (61.9%)	1.93 (0.88–4.21)	0.099
Hyperlipidemia				
No	41 (58.6%)	25 (59.5%)		
Yes	29 (41.4%)	17 (40.5%)	0.96 (0.44–2.09)	0.921
Coronary artery disease				
No	57 (81.4%)	29 (69.0%)		
Yes	13 (18.6%)	13 (31.0%)	1.97 (0.81–4.78)	0.136
Atrial fibrillation				
No	67 (95.7%)	38 (90.5%)		
Yes	3 (4.3%)	4 (9.5%)	2.35 (0.50–11.06)	0.279
Prior stroke				
No	50 (71.4%)	33 (78.6%)		
Yes	20 (28.6%)	9 (21.4%)	0.68 (0.28–1.68)	0.405
Stenosis location			2.17 (0.40–11.60)	0.366
ICA	26 (37.1%)	9 (21.4%)		
MCA	40 (57.1%)	30 (71.4%)		
ICA + MCA	4 (5.7%)	3 (7.1%)		
Medication use				
Antihypertensive drugs			0.45 (0.11, 1.77)	0.253
No	4 (5.7%)	5 (11.9%)		
Yes	66 (94.3%)	37 (88.1%)		
Lipid-lowering drugs			2.49 (0.27, 23.01)	0.423
No	4 (5.7%)	1 (2.4%)		
Yes	66 (94.3%)	41 (97.6%)		
Antiplatelet agents			0.73 (0.18, 2.89)	0.655
No	5 (7.1%)	4 (9.5%)		
Yes	65 (92.9%)	38 (90.5%)		
Drugs for microcirculation improvement			0.59 (0.26, 1.32)	0.197
No	20 (28.6%)	17 (40.5%)		
Yes	50 (71.4%)	25 (59.5%)		
DWI-ASPECTS				
≤ 7	34 (48.6%)	26 (61.9%)		
> 7	36 (51.4%)	16 (38.1%)	0.58 (0.27–1.27)	0.172
FVH score				
≤ 2.5	49 (70.0%)	8 (19.0%)		
> 2.5	21 (30.0%)	34 (81.0%)	9.92 (3.93–24.99)	<0.001
rLMC score				
≤ 11.5	13 (18.6%)	30 (71.4%)		
> 11.5	57 (81.4%)	12 (28.6%)	0.09 (0.04–0.22)	<0.001
Maas score				
≤ 2	17 (24.3%)	29 (69.0%)		
> 2	53 (75.7%)	13 (31.0%)	0.14 (0.06–0.34)	<0.001
ASITN score	2.9 ± 0.8	3.1 ± 1.1	1.31 (0.86–2.00)	0.214
rCBV				
> 1.11	40 (57.1%)	8 (19.0%)		
≤ 1.11	30 (42.9%)	34 (81.0%)	5.67 (2.29–13.99)	<0.001
rCBF				
≤ 0.97	31 (44.3%)	32 (76.2%)		
> 0.97	39 (55.7%)	10 (23.8%)	0.25 (0.11–0.58)	0.001
Systolic BP (mmHg)	147.2 ± 22.0	151.0 ± 23.0	1.01 (0.99–1.03)	0.384
Diastolic BP (mmHg)	87.5 ± 13.3	83.4 ± 15.9	0.98 (0.95–1.01)	0.145
Heart rate (bpm)	79.4 ± 12.1	78.8 ± 14.6	1.00 (0.97–1.03)	0.806
Total cholesterol (mmol/L)	8.8 ± 36.8	4.3 ± 1.0	0.90 (0.62–1.29)	0.554
Triglycerides (mmol/L)	1.9 ± 1.3	1.8 ± 1.0	0.88 (0.62–1.24)	0.45
HDL (mmol/L)	1.0 ± 0.3	1.0 ± 0.2	1.28 (0.26–6.37)	0.762
LDL (mmol/L)	2.7 ± 0.9	2.6 ± 0.8	0.88 (0.57–1.35)	0.554
Uric acid (μmol/L)	318.6 ± 88.1	304.1 ± 76.8	1.00 (0.99–1.00)	0.377
Homocysteine (μmol/L)	13.3 ± 7.6	13.4 ± 7.2	1.00 (0.95–1.06)	0.912
Blood glucose (mmol/L)	7.1 ± 2.9	8.0 ± 3.6	1.09 (0.97–1.23)	0.137
HbA1c (%)	6.9 ± 1.6	7.2 ± 1.2	1.18 (0.90–1.55)	0.239

NIHSS, National Institutes of Health Stroke Scale; ICA, internal carotid artery; MCA, middle cerebral artery; DWI-ASPECTS, diffusion-weighted imaging–Alberta Stroke Program Early CT Score; FVH, FLAIR vascular hyperintensity; rLMC, regional leptomeningeal collaterals; Maas, Maasricht Acute Stroke Score; ASITN/SIR, American Society of Interventional and Therapeutic Neuroradiology/Society of Interventional Radiology; rCBV, relative cerebral blood volume; rCBF, relative cerebral blood flow; HDL, high-density lipoprotein; LDL, low-density lipoprotein.

A multivariate binary logistic regression model was performed to identify independent predictors of poor functional outcome. The final model included all key imaging and hemodynamic parameters of interest. To control for potential confounding, the following clinically important baseline variables were forced into the model regardless of their univariate significance: age, history of atrial fibrillation, history of diabetes, and admission blood glucose level. Variable selection was based on both statistical significance (*p* < 0.1 in univariate analysis) and clinical relevance. The results are presented as adjusted odds ratios (ORs) with 95% confidence intervals (CIs).

Multivariate logistic regression analysis was performed to evaluate the independent associations between various factors and poor functional outcomes, after adjusting for clinical covariates. Such as age, history of diabetes, history of atrial fibrillation, and blood glucose levels. The results indicated that a higher admission National Institutes of Health Stroke Scale score (>3), a higher fluid-attenuated inversion recovery vascular hyperintensity score (>2.5), and a lower relative cerebral blood volume (≤1.11) were independent risk factors for poor 90-day functional outcomes. Their adjusted odds ratios (95% confidence intervals) were 4.01 (1.10–14.62), 6.35 (1.81–22.33), and 4.52 (1.12–18.20), respectively. Conversely, a higher regional leptomeningeal collateral score (>11) was a significant independent protective factor, with an adjusted odds ratio of 0.12 (0.02–0.63). All these associations were statistically significant (all *p*-values < 0.05).

In the adjusted model, no statistically significant associations were found between poor outcomes and age, history of diabetes, history of atrial fibrillation, blood glucose, Maas score (>2), or relative cerebral blood flow (>0.97) (all *p*-values > 0.05). The detailed results of the analysis are summarized in (Table 5).

**Table 5:** Multivariate logistic regression analysis for poor outcome, adjusted for clinical covariates.

Variable	Adjusted OR(95%CI)	*p*-value
Age	1.04 (0.98–1.10)	0.206
Diabetes	1.70 (0.39–7.31)	0.477
Fibrillation	3.43 (0.44–26.54)	0.237
Blood glucose	1.07 (0.84–1.35)	0.594
NIHSS score > 3.5	4.01 (1.10–14.62)	**0.035**
FVH score > 2.5	6.35 (1.81–22.33)	**0.004**
rLMC score > 11.5	0.12 (0.02–0.63)	**0.012**
Maas score > 2	0.61 (0.11–3.22)	0.557
rCBV ≤ 1.11	4.52 (1.12–18.20)	**0.034**
rCBF > 0.97	0.29 (0.07–1.24)	0.096

SE, standard error; OR, odds ratio; CI, confidence interval; NIHSS, National Institutes of Health Stroke Scale; FVH, FLAIR vascular hyperintensity; rLMC, regional leptomeningeal collaterals; Maas, Maasricht Acute Stroke Score; ASITN/SIR, American Society of Interventional and Therapeutic Neuroradiology/Society of Interventional Radiology; rCBV, relative cerebral blood volume; rCBF, relative cerebral blood flow. Bold values indicate statistically significant predictors (*p* < 0.05).

### 3.6 ROC curve analysis of factors associated with poor prognosis

**Figure 4:**
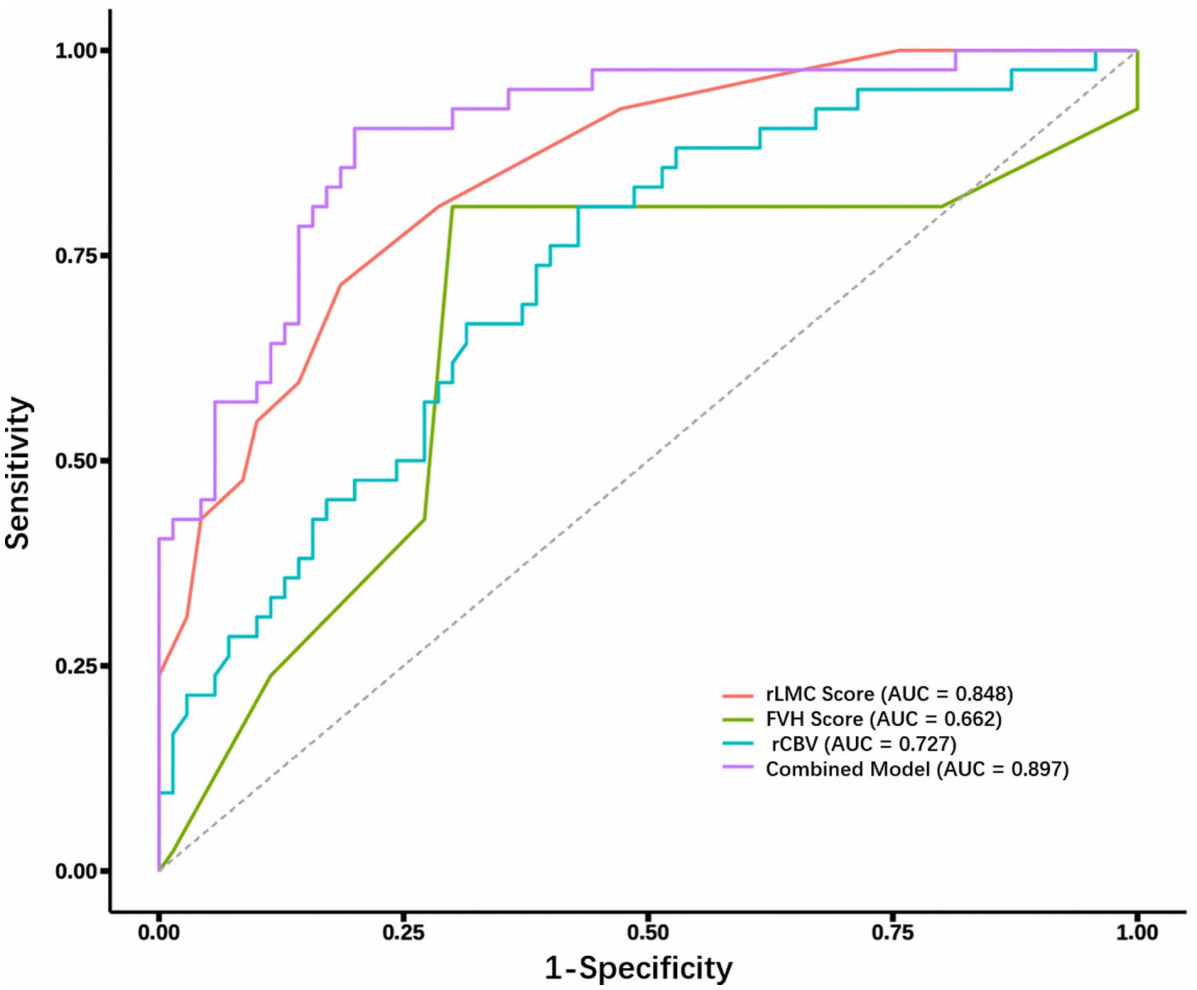
ROC analysis of FVH, rLMC, and rCBV individually and in combination for predicting poor outcomes. ROC, receiver operating characteristic; FVH, FLAIR vascular hyperintensity; rLMC, regional leptomeningeal collaterals; rCBV, relative cerebral blood volume; AUC, area under the curve.

ROC curve analysis was performed to evaluate the predictive efficacy of individual parameters (NIHSS score, FVH score, rLMC score, rCBV) and a combined model (FVH + rLMC + rCBV) for poor 90-day functional outcomes (Figure 4).

Among the individual indicators, the rLMC score demonstrated the highest predictive value with an AUC of 0.848, followed by rCBV (AUC = 0.727), admission NIHSS score (AUC = 0.727), and FVH score (AUC = 0.662). The optimal cut-off values for these parameters were: rLMC score ≤11.5 (sensitivity 0.814, specificity 0.714), NIHSS score >3.5 (sensitivity 0.667, specificity 0.686), rCBV ≤1.111 (sensitivity 0.571, specificity 0.810), and FVH score >2.5 (sensitivity 0.810, specificity 0.700). The rLMC score also yielded the highest Youden’s index (0.528) among the individual variables. In contrast, the combined prediction model integrating FVH, rLMC, and rCBV exhibited superior discriminatory power, achieving a significantly higher AUC of 0.897 (95% CI: 0.837–0.956) and the highest overall Youden’s index (0.610), with balanced sensitivity (80.0%) and specificity (81.0%) (Table 6).

**Table 6:** The area under the ROC curve (AUC) of various parameters for predicting poor prognosis in AIS.

Variable	AUC (95% CI)	Sensitivity (95% CI)	Specificity (95% CI)	Cut-off	Youden’s Index
NIHSS score	0.727 (0.628–0.826)	0.667 (0.524–0.809)	0.686 (0.577–0.794)	>3.5	0.353
FVH score	0.662 (0.550–0.774)	0.810 (0.691–0.928)	0.700 (0.593–0.807)	>2.5	0.51
rLMC score	0.848 (0.778–0.919)	0.814 (0.723–0.905)	0.714 (0.578–0.851)	≤11.5	0.528
rCBV	0.727 (0.631–0.822)	0.571 (0.455–0.687)	0.810 (0.691–0.928)	≤1.111	0.381
Combined prediction model	0.897 (0.837–0.956)	0.800 (0.706–0.894)	0.810 (0.706–0.933)	0.816	0.610

NIHSS, National Institutes of Health Stroke Scale; FVH, FLAIR vascular hyperintensity; rLMC, regional leptomeningeal collaterals; rCBV, relative cerebral blood volume; AUC, area under the curve; CI, confidence interval. The combined model incorporates FVH score, rLMC score, and rCBV.

## 4 Discussion

Intravenous thrombolysis remains the most effective pharmacological treatment for improving outcomes in acute ischemic stroke. However, the clinical application of the most commonly used thrombolytic agent, alteplase, is limited by its narrow therapeutic window of 4.5 h (18), while urokinase, now less frequently used, has a slightly extended but still restrictive 6-h window (19). Various factors often prevent patients from receiving timely thrombolytic therapy within these critical timeframes, significantly constraining its clinical utility. Recent clinical studies have demonstrated that robust collateral circulation can effectively limit the expansion of the infarct core, enhance clinical outcomes, and reduce the risk of disease recurrence (20). Consequently, the establishment of collateral circulation, particularly through leptomeningeal arteries, has emerged as a crucial independent factor influencing the prognosis of ischemic stroke patients (21).

Collateral circulation refers to the anastomotic vascular structures connecting adjacent arterial branches, present in most human tissues. Under physiological conditions, these structures regulate blood flow to meet tissue metabolic demands. In pathological scenarios such as severe stenosis or occlusion of major arteries, these anastomotic channels can establish alternative blood flow pathways to maintain perfusion to ischemic tissues (22).

In previous research, the ASITN/SIR scoring system has been established as the gold standard for cerebrovascular assessment due to its internationally validated reliability through multicenter clinical trials (17, 23). This system combines the high spatial resolution of digital subtraction angiography (capable of visualizing even the smallest leptomeningeal vessels) with real-time dynamic imaging, providing clinicians with comprehensive multidimensional information about blood flow patterns, velocity, and vascular morphology (24). Although the traditional ASITN/SIR grading system—the benchmark for global collateral assessment—failed to significantly distinguish between the prognostic groups (which might be attributed to the predominantly intermediate-grade collateral status in our conservatively treated cohort), this finding indicates that while the ASITN/SIR system provides a valid macroscopic overview of collateral status, it may lack the sensitivity to detect more subtle, regionally heterogeneous differences in collateral perfusion, which are crucial for predicting outcomes in patients managed conservatively. Our study evaluated the correlations between rLMC score, Maas score, FVH score, and ASITN/SIR grading. The experimental data demonstrated that the rLMC score showed the highest correlation coefficient with ASITN/SIR grading (*p* < 0.001) compared to other scoring systems.

Numerous studies have indicated that FVH formation is closely associated with leptomeningeal collateral compensation (9, 11, 25, 26). The rLMC score quantitatively assesses collateral circulation function by evaluating the extent of collateral vessel recruitment and compensatory range. Our findings are consistent with the concept that the FVH score may provide a quantitative measure of collateral compensation capacity. This correlation may exist because FVH scoring, by counting hyperintense vessels on FLAIR sequences, indirectly reflects the total number of recruited collateral vessels during acute ischemia. Furthermore, the hyperintensity resulting from absent flow voids due to sluggish blood flow may correlate with the severity of hemodynamic impairment. Similarly, CTA-based collateral grading evaluates visible collateral vessels, providing a numerical assessment of collateral function from a different imaging modality. When leptomeningeal collaterals are partially recruited (low rLMC score) but fail to maintain effective perfusion due to increased microvascular resistance or blood–brain barrier disruption, FVH may indicate unsuccessful compensation attempts rather than true reperfusion.

This retrospective cohort study observed a moderate inverse correlation between FLAIR-based FVH scores and CTA collateral assessments, which could reflect the differential sensitivity of these modalities to distinct compensatory phases of collateral circulation. The hemodynamic basis of FVH was supported by its consistent localization with vascular occlusion territories. FVH is considered an imaging marker of cerebral hypoperfusion while potentially, indirectly mirroring the status of collateral flow pathways. Furthermore, beyond collateral grading, FVH is likely influenced by other factors that should be considered in future neuroimaging models of stroke outcomes.

Our study systematically analyzed key factors affecting outcomes in conservatively treated acute ischemic stroke (AIS) patients by integrating multimodal imaging parameters and clinical indicators. The results revealed that patients with poor outcomes typically exhibited: (1) significantly higher admission NIHSS scores; (2) more extensive FVH hyperintensity on FLAIR sequences; (3) markedly reduced rLMC collateral scores on CTA-CTP; and (4) significantly decreased cerebral blood volume (CBV) values in affected regions.

The admission NIHSS score emerged as an independent predictor of AIS patient outcomes, with higher scores associated with poorer prognosis. Widely used in clinical practice, NIHSS provides a preliminary assessment of neurological function, which is closely related to perfusion compensation in ischemic regions – a key determinant of patient outcomes. This finding aligns with previous studies identifying admission NIHSS as an independent prognostic factor for AIS patients (22, 27).

The rLMC score, a quantitative CTA-based evaluation method, offers comprehensive coverage of six ASPECTS regions in the middle cerebral artery territory, anterior cerebral artery region, and basal ganglia, with particular emphasis on sylvian fissure artery scoring (0/2/4 points) (16, 28). Compared to other collateral scoring systems (such as Tan score or Maas score), rLMC provides a more detailed regional assessment, better reflecting collateral compensation after large vessel occlusion. The degree of collateral compensation in these regions significantly correlates with reduced infarct volume (29). Our multimodal imaging analysis suggested that a higher regional leptomeningeal collateral (rLMC) score was independently associated with 90-day functional outcomes in patients with acute ischemic stroke (AIS). Specifically, multivariate logistic regression indicated that patients with an rLMC score > 11.5 had a significantly lower risk of poor outcomes (OR = 0.12, 95% CI 0.02–0.63, *p* = 0.012), consistent with Menon et al.’s findings (16), while extending clinical applications through combination with FVH and CTP parameters.

The pathophysiological mechanisms of FVH remain an area of active investigation, with interpretations appearing to be context-dependent. Generally attributed to slow flow distal to a large vessel occlusion, the clinical significance of FVH may vary with factors such as occlusion severity, collateral status, and, notably, the treatment strategy employed (e.g., reperfusion therapy versus conservative management) (30). Evidence suggests that the prognostic value of FVH is significantly influenced by the time elapsed from symptom onset to imaging acquisition. Studies reporting an association between FVH and favorable outcomes predominantly involve patients imaged within a very early time window (e.g., <6 h), often coinciding with the therapeutic window for reperfusion (26, 31). In this specific context, FVH is frequently interpreted as a radiographic marker of potentially salvageable tissue via active leptomeningeal collaterals. Consequently, a positive FVH status at this hyperacute stage, particularly when followed by successful recanalization, is frequently associated with better outcomes, as the underlying ischemic penumbra may be reversed. In contrast, the present study, along with other reports linking FVH to poor outcomes, typically involves patients assessed at later time points (12–24 h or longer). Our cohort is characterized by the absence of reperfusion therapy and a broader imaging window (up to 72 h). Here, a higher FVH score was independently associated with poor 90-day outcomes (14, 25, 32, 33), while other studies have reported that FVH has limited independent prognostic value (34). This variability in findings may stem, in part, from the established association between extensive FVH and the presence of severe proximal arterial stenosis or occlusion (31). Our multivariate logistic regression analysis indicated that a higher FVH score was an independent factor associated with poor 90-day functional outcomes, alongside other imaging parameters. This observation, reinforced by ROC curve analysis confirming FVH’s predictive performance, suggests a distinct interpretation in the context of persistent large vessel occlusion without reperfusion therapy. Beyond the acute therapeutic window, prominent FVH may not merely signify recruited collateral flow but could rather indicate a state of insufficient collateral compensation that is inadequate for long-term tissue maintenance. This interpretation aligns with the observed negative correlation between FVH scores and robust collateral grades, and it is further supported by its association with unfavorable perfusion parameters. Consequently, for patients managed conservatively, as in the present cohort, a higher FVH-ASPECT score appears to identify a subgroup of patients with a significant association to poorer functional outcomes despite standard medical therapy. In summary, pretreatment FVH assessment provides valuable information for predicting functional outcomes in AIS patients not undergoing reperfusion therapy. The variable prognostic significance of FVH underscores the importance of considering both the temporal evolution of the ischemic cascade and the treatment strategy applied when interpreting this imaging finding.

In our study, rCBV emerged as another independent predictor of 90-day poor outcomes. Results showed significantly higher rCBV values in the favorable outcome group (mRS 0–2) compared to the poor outcome group (mRS 3–6) (*p* < 0.05), confirmed by multivariate logistic regression. This finding aligns with previous research (35, 36), as CBV closely relates to collateral status and reflects compensatory capacity for hemodynamic changes. CBV serves as a crucial parameter for assessing whether cerebral autoregulation remains functional after ischemia (37, 38). Reduced CBV typically indicates microcirculatory impairment in local brain tissue. In AIS caused by hypoperfusion-induced cerebral hypoxia, higher CBV values suggest adequate tissue oxygenation, and a lower risk of microcirculatory dysfunction affects prognosis (39). Significant CBV reduction effectively identifies and evaluates stroke severity, particularly in distinguishing normal from ischemic brain tissue (40). Our findings confirm the association between reduced rCBV and poor outcomes, while demonstrating the synergistic value of combining FVH scoring with multimodal CT parameters (rLMC score, rCBV) for predicting collateral status and functional outcomes.

The combined prediction model demonstrated significantly superior predictive power (AUC = 0.90) compared to any individual imaging parameter. This integration of multimodal data effectively overcomes the inherent limitations of single-modality assessment. The combined prediction model constructed in this study incorporated only imaging parameters (FVH, rLMC, and rCBV), with the aim of focusing on the independent and combined value of these radiological biomarkers. Although the admission NIHSS score is an important clinical prognostic indicator, its nature and origin as a clinical functional assessment tool differ from those of imaging parameters. Limiting the model to imaging indicators facilitates the provision of an imaging-based decision-making tool for clinical use that does not rely on immediate neurological function scores. Consequently, the model offers a robust and reliable tool for clinical decision-making and prognosis estimation in acute ischemic stroke. The findings of this study indicate potential for clinical translation. The imaging sequences (FLAIR, CTA–CTP) required for the combined model are already part of the standard protocol in most stroke centers, indicating their potential for high applicability in resource-limited environments. The cutoff values revealed by the research offer a possible objective basis for clinical triage. More concretely, the model may help identify patients who have a non-severe NIHSS score at admission but carry a potential risk of poor prognosis due to poor collateral circulation shown by imaging. For this patient group, clinical decisions could contemplate providing a higher level of medical care, which might contribute to a more precise allocation of limited medical resources to this high-risk group and provide a reference for resource management.

This study has several limitations. First, as a single-center retrospective analysis, the generalizability of its conclusions requires validation in larger cohorts, and the relatively modest sample size may constrain statistical power. Second, although the exclusion of patients who received reperfusion therapy and the 72-h onset-to-imaging window served to clarify the relationship between imaging biomarkers and the natural history of stroke, these design choices may limit the direct applicability of the findings to broader therapeutic contexts. Furthermore, in terms of imaging assessment, factors such as the time-dependent evolution of FVH, non-simultaneous acquisition of CT and MRI scans, and unmeasured physiological confounders may have introduced additional heterogeneity and bias. Future multicenter, prospective studies incorporating stricter imaging protocols and comprehensive physiological monitoring are warranted to accurately validate the prognostic value of these imaging biomarkers.

## 5 Conclusion

In conclusion, admission NIHSS score, FVH score, rLMC score, and rCBV may serve as independent predictors for conservative treatment outcomes in AIS patients, with FVH score negatively correlating with collateral scores. Specifically, high FVH scores combined with low rLMC scores and low rCBV values correlate with poor outcomes, and their combined use enhances prediction accuracy. The integration of multimodal CT and MRI facilitates early AIS diagnosis and comprehensive collateral circulation assessment.

## Data Availability

The raw data supporting the conclusions of this article will be made available by the authors, without undue reservation.
